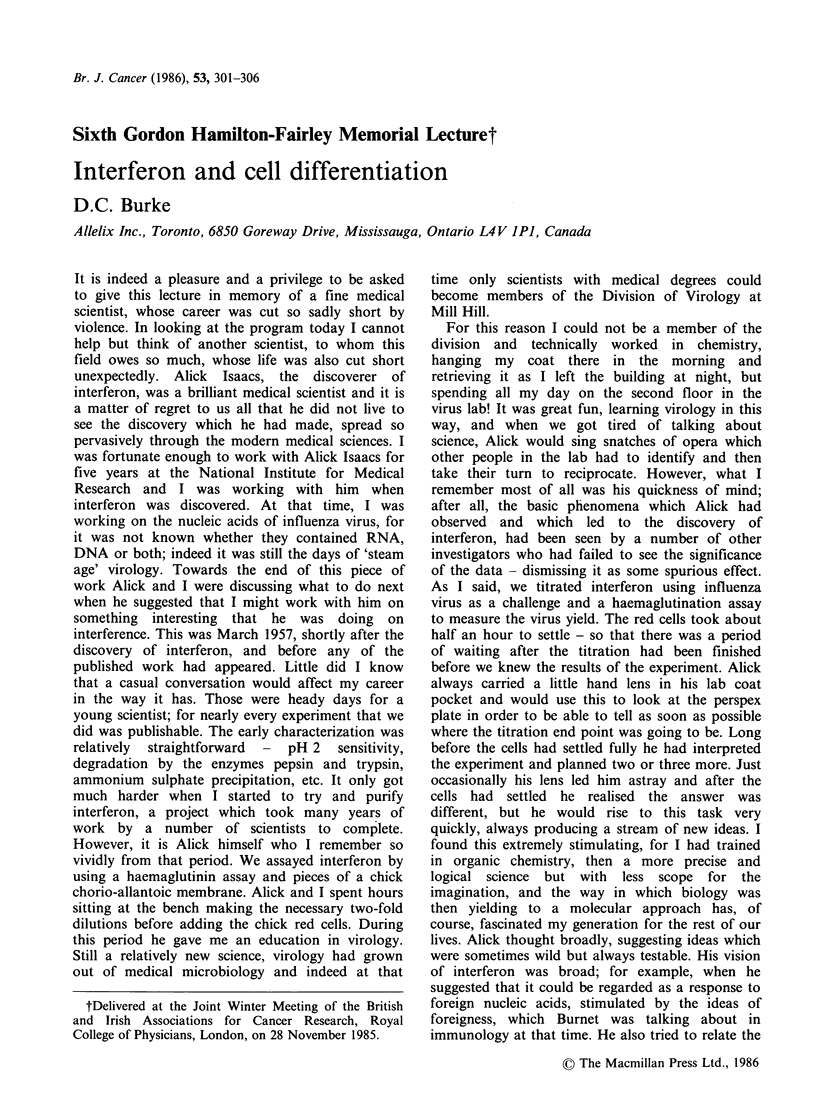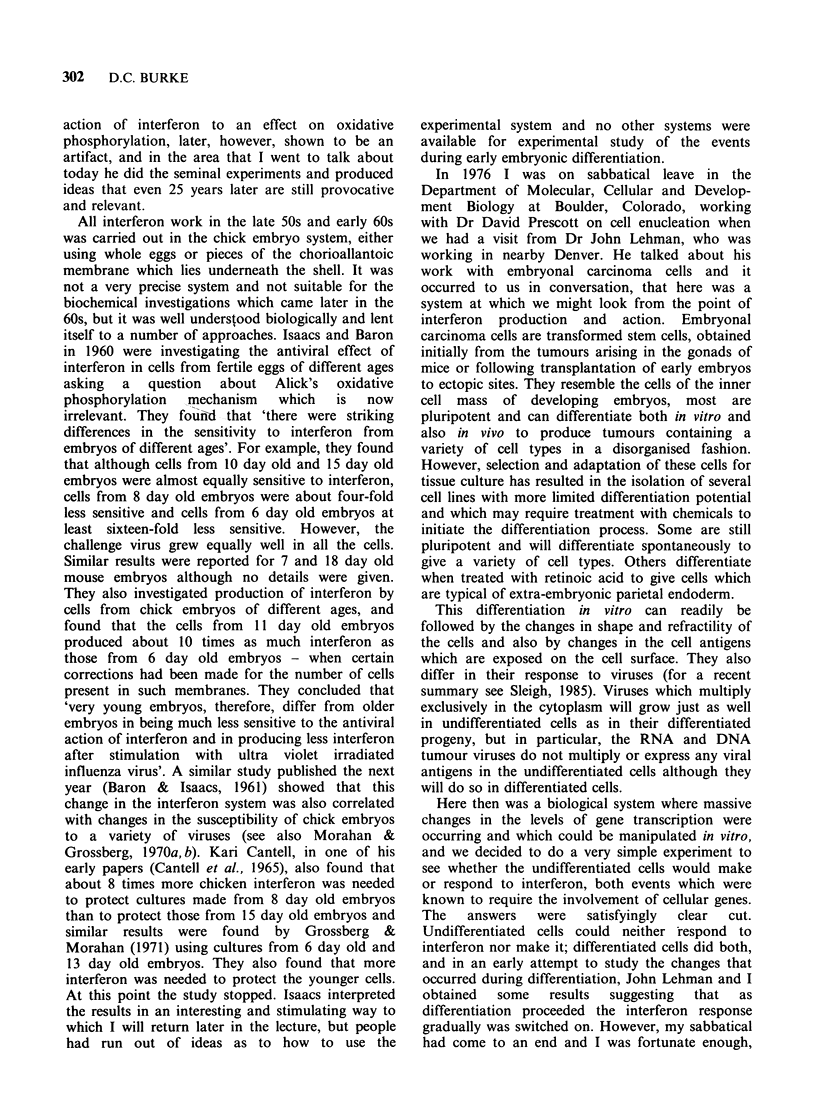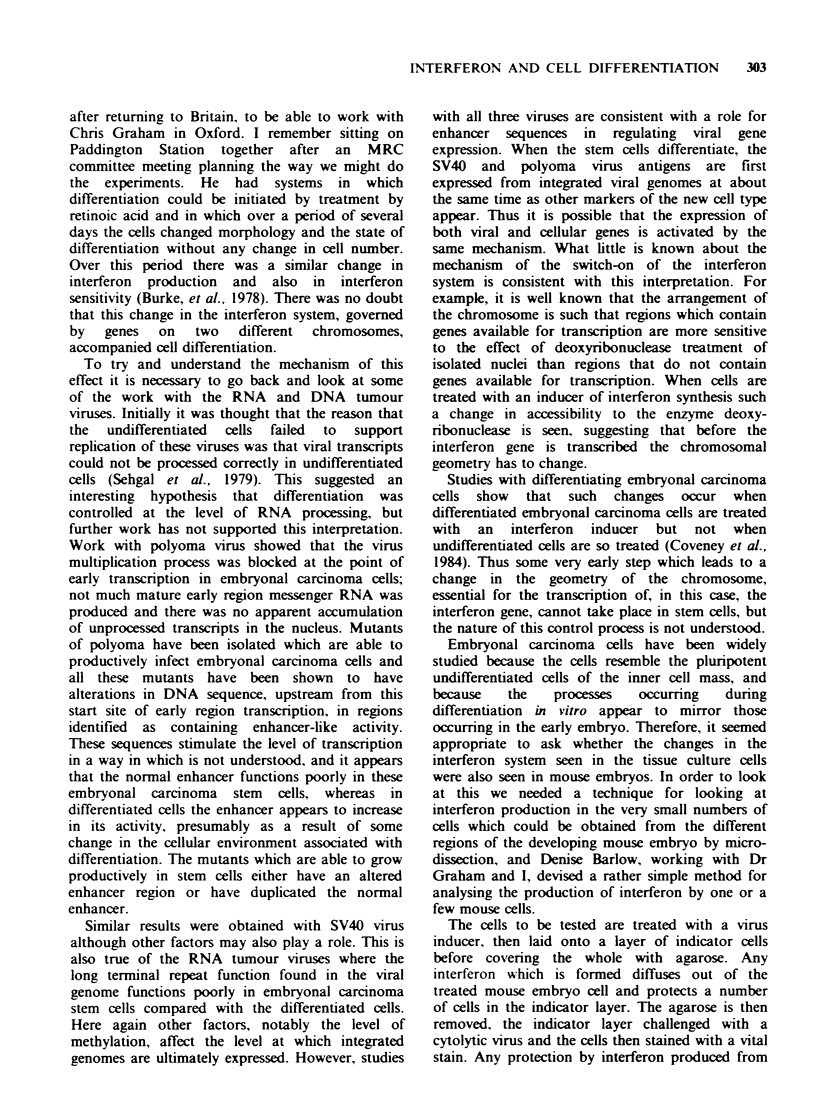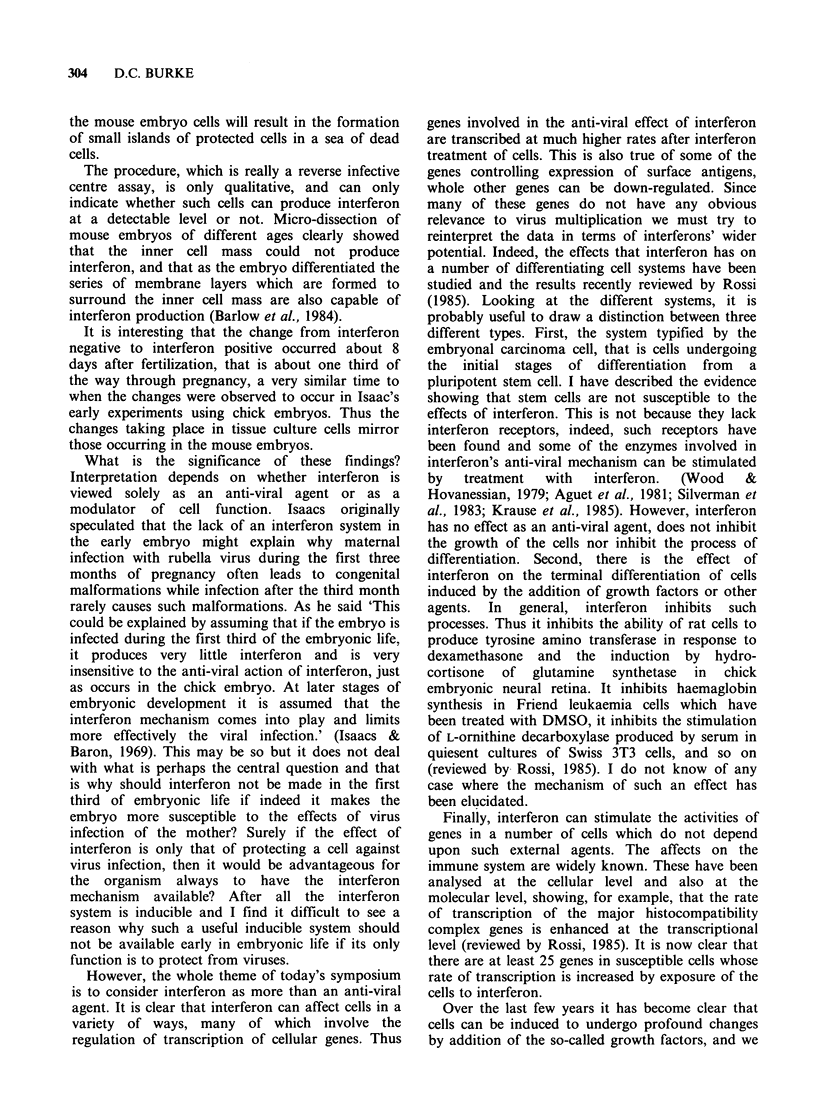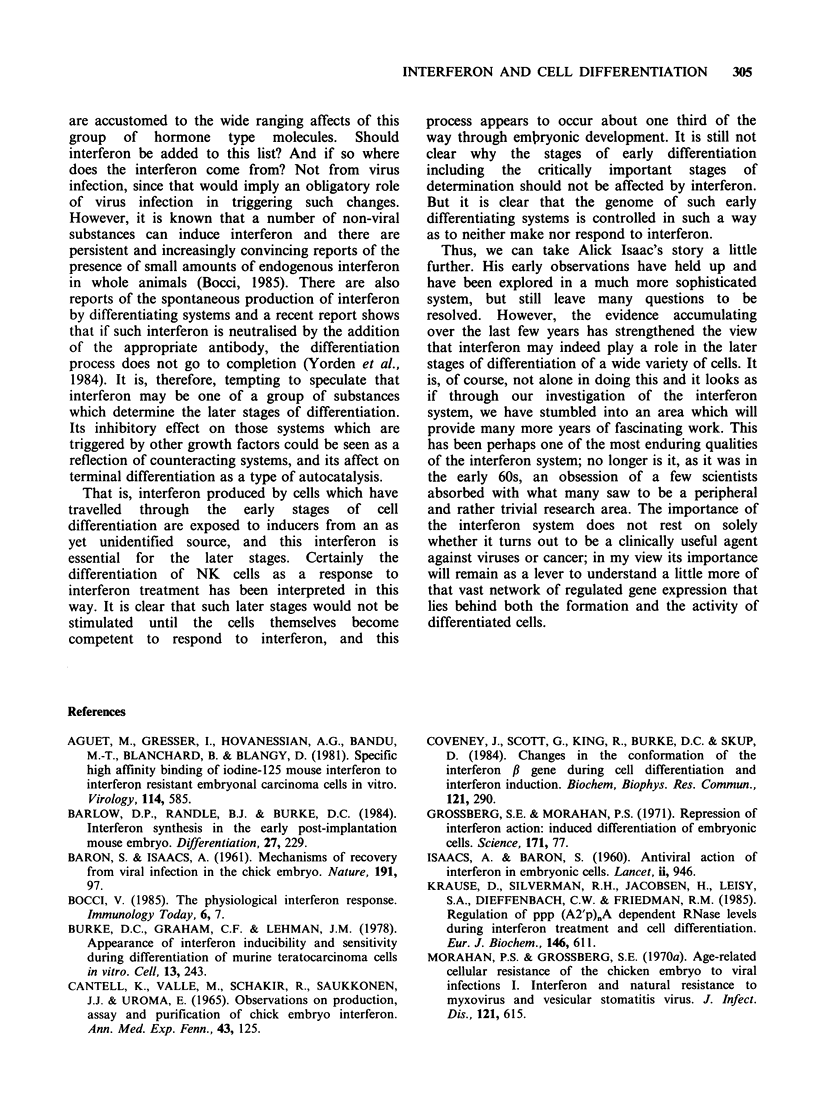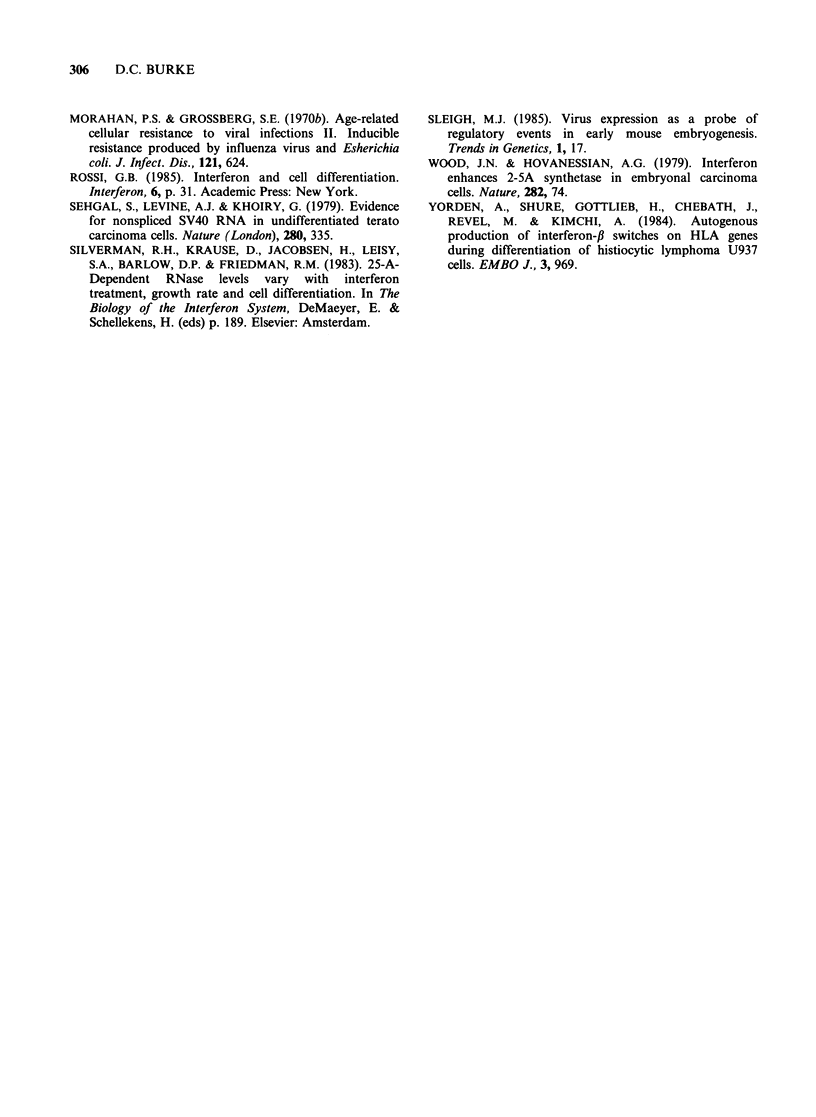# Interferon and cell differentiation.

**DOI:** 10.1038/bjc.1986.52

**Published:** 1986-03

**Authors:** D. C. Burke


Br. J. Cancer (1986), 53, 301–306

### Sixth Gordon Hamilton-Fairley Memorial Lecture†

### Interferon and cell differentiation

D.C. Burke

*Allelix Inc., Toronto, 6850 Goreway Drive, Mississauga, Ontario L4V 1P1, Canada*

It is indeed a pleasure and a privilege to be asked to give this lecture in memory of a fine medical scientist, whose career was cut so sadly short by violence. In looking at the program today I cannot help but think of another scientist, to whom this field owes so much, whose life was also cut short unexpectedly. Alick Isaacs, the discoverer of interferon, was a brilliant medical scientist and it is a matter of regret to us all that he did not live to see the discovery which he had made, spread so pervasively through the modern medical sciences. I was fortunate enough to work with Alick Isaacs for five years at the National Institute for Medical Research and I was working with him when interferon was discovered. At that time, I was working on the nucleic acids of influenza virus, for it was not known whether they contained RNA, DNA or both; indeed it was still the days of 'steam age' virology. Towards the end of this piece of work Alick and I were discussing what to do next when he suggested that I might work with him on something interesting that he was doing on interference. This was March 1957, shortly after the discovery of interferon, and before any of the published work had appeared. Little did I know that a casual conversation would affect my career in the way it has. Those were heady days for a young scientist; for nearly every experiment that we did was publishable. The early characterization was relatively straightforward – pH 2 sensitivity, degradation by the enzymes pepsin and trypsin, ammonium sulphate precipitation, etc. It only got much harder when I started to try and purify interferon, a project which took many years of work by a number of scientists to complete. However, it is Alick himself who I remember so vividly from that period. We assayed interferon by using a haemaglutinin assay and pieces of a chick chorio-allantoic membrane. Alick and I spent hours sitting at the bench making the necessary two-fold dilutions before adding the chick red cells. During this period he gave me an education in virology. Still a relatively new science, virology had grown out of medical microbiology and indeed at that time only scientists with medical degrees could become members of the Division of Virology at Mill Hill.

For this reason I could not be a member of the division and technically worked in chemistry, hanging my coat there in the morning and retrieving it as I left the building at night, but spending all my day on the second floor in the virus lab! It was great fun, learning virology in this way, and when we got tired of talking about science, Alick would sing snatches of opera which other people in the lab had to identify and then take their turn to reciprocate. However, what I remember most of all was his quickness of mind; after all, the basic phenomena which Alick had observed and which led to the discovery of interferon, had been seen by a number of other investigators who had failed to see the significance of the data – dismissing it as some spurious effect. As I said, we titrated interferon using influenza virus as a challenge and a haemaglutination assay to measure the virus yield. The red cells took about half an hour to settle – so that there was a period of waiting after the titration had been finished before we knew the results of the experiment. Alick always carried a little hand lens in his lab coat pocket and would use this to look at the perspex plate in order to be able to tell as soon as possible where the titration end point was going to be. Long before the cells had settled fully he had interpreted the experiment and planned two or three more. Just occasionally his lens led him astray and after the cells had settled he realised the answer was different, but he would rise to this task very quickly, always producing a stream of new ideas. I found this extremely stimulating, for I had trained in organic chemistry, then a more precise and logical science but with less scope for the imagination, and the way in which biology was then yielding to a molecular approach has, of course, fascinated my generation for the rest of our lives. Alick thought broadly, suggesting ideas which were sometimes wild but always testable. His vision of interferon was broad; for example, when he suggested that it could be regarded as a response to foreign nucleic acids, stimulated by the ideas of foreigness, which Burnet was talking about in immunology at that time. He also tried to relate the

†Delivered at the Joint Winter Meeting of the British and Irish Associations for Cancer Research, Royal College of Physicians, London, on 28 November 1985.

© The Macmillan Press Ltd., 1986

action of interferon to an effect on oxidative phosphorylation, later, however, shown to be an artifact, and in the area that I went to talk about today he did the seminal experiments and produced ideas that even 25 years later are still provocative and relevant.

All interferon work in the late 50s and early 60s was carried out in the chick embryo system, either using whole eggs or pieces of the chorioallantoic membrane which lies underneath the shell. It was not a very precise system and not suitable for the biochemical investigations which came later in the 60s, but it was well understood biologically and lent itself to a number of approaches. Isaacs and Baron in 1960 were investigating the antiviral effect of interferon in cells from fertile eggs of different ages asking a question about Alick's oxidative phosphorylation mechanism which is now irrelevant. They found that 'there were striking differences in the sensitivity to interferon from embryos of different ages'. For example, they found that although cells from 10 day old and 15 day old embryos were almost equally sensitive to interferon, cells from 8 day old embryos were about four-fold less sensitive and cells from 6 day old embryos at least sixteen-fold less sensitive. However, the challenge virus grew equally well in all the cells. Similar results were reported for 7 and 18 day old mouse embryos although no details were given. They also investigated production of interferon by cells from chick embryos of different ages, and found that the cells from 11 day old embryos produced about 10 times as much interferon as those from 6 day old embryos – when certain corrections had been made for the number of cells present in such membranes. They concluded that 'very young embryos, therefore, differ from older embryos in being much less sensitive to the antiviral action of interferon and in producing less interferon after stimulation with ultra violet irradiated influenza virus'. A similar study published the next year (Baron & Isaacs, 1961) showed that this change in the interferon system was also correlated with changes in the susceptibility of chick embryos to a variety of viruses (see also Morahan & Grossberg, 1970*a,b*). Kari Cantell, in one of his early papers (Cantell *et al.*, 1965), also found that about 8 times more chicken interferon was needed to protect cultures made from 8 day old embryos than to protect those from 15 day old embryos and similar results were found by Grossberg & Morahan (1971) using cultures from 6 day old and 13 day old embryos. They also found that more interferon was needed to protect the younger cells. At this point the study stopped. Isaacs interpreted the results in an interesting and stimulating way to which I will return later in the lecture, but people had run out of ideas as to how to use the experimental system and no other systems were available for experimental study of the events during early embryonic differentiation.

In 1976 I was on sabbatical leave in the Department of Molecular, Cellular and Development Biology at Boulder, Colorado, working with Dr David Prescott on cell enucleation when we had a visit from Dr John Lehman, who was working in nearby Denver. He talked about his work with embryonal carcinoma cells and it occurred to us in conversation, that here was a system at which we might look from the point of interferon production and action. Embryonal carcinoma cells are transformed stem cells, obtained initially from the tumours arising in the gonads of mice or following transplantation of early embryos to ectopic sites. They resemble the cells of the inner cell mass of developing embryos, most are pluripotent and can differentiate both *in vitro* and also *in vivo* to produce tumours containing a variety of cell types in a disorganised fashion. However, selection and adaptation of these cells for tissue culture has resulted in the isolation of several cell lines with more limited differentiation potential and which may require treatment with chemicals to initiate the differentiation process. Some are still pluripotent and will differentiate spontaneously to give a variety of cell types. Others differentiate when treated with retinoic acid to give cells which are typical of extra-embryonic parietal endoderm.

This differentiation *in vitro* can readily be followed by the changes in shape and refractility of the cells and also by changes in the cell antigens which are exposed on the cell surface. They also differ in their response to viruses (for a recent summary see Sleigh, 1985). Viruses which multiply exclusively in the cytoplasm will grow just as well in undifferentiated cells as in their differentiated progeny, but in particular, the RNA and DNA tumour viruses do not multiply or express any viral antigens in the undifferentiated cells although they will do so in differentiated cells.

Here then was a biological system where massive changes in the levels of gene transcription were occurring and which could be manipulated *in vitro*, and we decided to do a very simple experiment to see whether the undifferentiated cells would make or respond to interferon, both events which were known to require the involvement of cellular genes. The answers were satisfyingly clear cut. Undifferentiated cells could neither respond to interferon nor make it; differentiated cells did both, and in an early attempt to study the changes that occurred during differentiation, John Lehman and I obtained some results suggesting that as differentiation proceeded the interferon response gradually was switched on. However, my sabbatical had come to an end and I was fortunate enough, after returning to Britain, to be able to work with Chris Graham in Oxford. I remember sitting on Paddington Station together after an MRC committee meeting planning the way we might do the experiments. He had systems in which differentiation could be initiated by treatment by retinoic acid and in which over a period of several days the cells changed morphology and the state of differentiation without any change in cell number. Over this period there was a similar change in interferon production and also in interferon sensitivity (Burke, *et al.*, 1978). There was no doubt that this change in the interferon system, governed by genes on two different chromosomes, accompanied cell differentiation.

To try and understand the mechanism of this effect it is necessary to go back and look at some of the work with the RNA and DNA tumour viruses. Initially it was thought that the reason that the undifferentiated cells failed to support replication of these viruses was that viral transcripts could not be processed correctly in undifferentiated cells (Sehgal *et al.*, 1979). This suggested an interesting hypothesis that differentiation was controlled at the level of RNA processing, but further work has not supported this interpretation. Work with polyoma virus showed that the virus multiplication process was blocked at the point of early transcription in embryonal carcinoma cells; not much mature early region messenger RNA was produced and there was no apparent accumulation of unprocessed transcripts in the nucleus. Mutants of polyoma have been isolated which are able to productively infect embryonal carcinoma cells and all these mutants have been shown to have alterations in DNA sequence, upstream from this start site of early region transcription, in regions identified as containing enhancer-like activity. These sequences stimulate the level of transcription in a way in which is not understood, and it appears that the normal enhancer functions poorly in these embryonal carcinoma stem cells, whereas in differentiated cells the enhancer appears to increase in its activity, presumably as a result of some change in the cellular environment associated with differentiation. The mutants which are able to grow productively in stem cells either have an altered enhancer region or have duplicated the normal enhancer.

Similar results were obtained with SV40 virus although other factors may also play a role. This is also true of the RNA tumour viruses where the long terminal repeat function found in the viral genome functions poorly in embryonal carcinoma stem cells compared with the differentiated cells. Here again other factors, notably the level of methylation, affect the level at which integrated genomes are ultimately expressed. However, studies with all three viruses are consistent with a role for enhancer sequences in regulating viral gene expression. When the stem cells differentiate, the SV40 and polyoma virus antigens are first expressed from integrated viral genomes at about the same time as other markers of the new cell type appear. Thus it is possible that the expression of both viral and cellular genes is activated by the same mechanism. What little is known about the mechanism of the switch-on of the interferon system is consistent with this interpretation. For example, it is well known that the arrangement of the chromosome is such that regions which contain genes available for transcription are more sensitive to the effect of deoxyribonuclease treatment of isolated nuclei than regions that do not contain genes available for transcription. When cells are treated with an inducer of interferon synthesis such a change in accessibility to the enzyme deoxyribonuclease is seen, suggesting that before the interferon gene is transcribed the chromosomal geometry has to change.

Studies with differentiating embryonal carcinoma cells show that such changes occur when differentiated embryonal carcinoma cells are treated with an interferon inducer but not when undifferentiated cells are so treated (Coveney *et al.*, 1984). Thus some very early step which leads to a change in the geometry of the chromosome, essential for the transcription of, in this case, the interferon gene, cannot take place in stem cells, but the nature of this control process is not understood.

Embryonal carcinoma cells have been widely studied because the cells resemble the pluripotent undifferentiated cells of the inner cell mass, and because the processes occurring during differentiation *in vitro* appear to mirror those occurring in the early embryo. Therefore, it seemed appropriate to ask whether the changes in the interferon system seen in the tissue culture cells were also seen in mouse embryos. In order to look at this we needed a technique for looking at interferon production in the very small numbers of cells which could be obtained from the different regions of the developing mouse embryo by microdissection, and Denise Barlow, working with Dr Graham and I, devised a rather simple method for analysing the production of interferon by one or a few mouse cells.

The cells to be tested are treated with a virus inducer, then laid onto a layer of indicator cells before covering the whole with agarose. Any interferon which is formed diffuses out of the treated mouse embryo cell and protects a number of cells in the indicator layer. The agarose is then removed, the indicator layer challenged with a cytolytic virus and the cells then stained with a vital stain. Any protection by interferon produced from the mouse embryo cells will result in the formation of small islands of protected cells in a sea of dead cells.

The procedure, which is really a reverse infective centre assay, is only qualitative, and can only indicate whether such cells can produce interferon at a detectable level or not. Micro-dissection of mouse embryos of different ages clearly showed that the inner cell mass could not produce interferon, and that as the embryo differentiated the series of membrane layers which are formed to surround the inner cell mass are also capable of interferon production (Barlow *et al.*, 1984).

It is interesting that the change from interferon negative to interferon positive occurred about 8 days after fertilization, that is about one third of the way through pregnancy, a very similar time to when the changes were observed to occur in Isaac's early experiments using chick embryos. Thus the changes taking place in tissue culture cells mirror those occurring in the mouse embryos.

What is the significance of these findings? Interpretation depends on whether interferon is viewed solely as an anti-viral agent or as a modulator of cell function. Isaacs originally speculated that the lack of an interferon system in the early embryo might explain why maternal infection with rubella virus during the first three months of pregnancy often leads to congenital malformations while infection after the third month rarely causes such malformations. As he said 'This could be explained by assuming that if the embryo is infected during the first third of the embryonic life, it produces very little interferon and is very insensitive to the anti-viral action of interferon, just as occurs in the chick embryo. At later stages of embryonic development it is assumed that the interferon mechanism comes into play and limits more effectively the viral infection.' (Isaacs & Baron, 1969). This may be so but it does not deal with what is perhaps the central question and that is why should interferon not be made in the first third of embryonic life if indeed it makes the embryo more susceptible to the effects of virus infection of the mother? Surely if the effect of interferon is only that of protecting a cell against virus infection, then it would be advantageous for the organism always to have the interferon mechanism available? After all the interferon system is inducible and I find it difficult to see a reason why such a useful inducible system should not be available early in embryonic life if its only function is to protect from viruses.

However, the whole theme of today's symposium is to consider interferon as more than an anti-viral agent. It is clear that interferon can affect cells in a variety of ways, many of which involve the regulation of transcription of cellular genes. Thus genes involved in the anti-viral effect of interferon are transcribed at much higher rates after interferon treatment of cells. This is also true of some of the genes controlling expression of surface antigens, whole other genes can be down-regulated. Since many of these genes do not have any obvious relevance to virus multiplication we must try to reinterpret the data in terms of interferons' wider potential. Indeed, the effects that interferon has on a number of differentiating cell systems have been studied and the results recently reviewed by Rossi (1985). Looking at the different systems, it is probably useful to draw a distinction between three different types. First, the system typified by the embryonal carcinoma cell, that is cells undergoing the initial stages of differentiation from a pluripotent stem cell. I have described the evidence showing that stem cells are not susceptible to the effects of interferon. This is not because they lack interferon receptors, indeed, such receptors have been found and some of the enzymes involved in interferon's anti-viral mechanism can be stimulated by treatment with interferon. (Wood & Hovanessian, 1979; Aguet *et al.*, 1981; Silverman *et al.*, 1983; Krause *et al.*, 1985). However, interferon has no effect as an anti-viral agent, does not inhibit the growth of the cells nor inhibit the process of differentiation. Second, there is the effect of interferon on the terminal differentiation of cells induced by the addition of growth factors or other agents. In general, interferon inhibits such processes. Thus it inhibits the ability of rat cells to produce tyrosine amino transferase in response to dexamethasone and the induction by hydrocortisone of glutamine synthetase in chick embryonic neural retina. It inhibits haemaglobin synthesis in Friend leukaemia cells which have been treated with DMSO, it inhibits the stimulation of L-ornithine decarboxylase produced by serum in quiesent cultures of Swiss 3T3 cells, and so on (reviewed by Rossi, 1985). I do not know of any case where the mechanism of such an effect has been elucidated.

Finally, interferon can stimulate the activities of genes in a number of cells which do not depend upon such external agents. The affects on the immune system are widely known. These have been analysed at the cellular level and also at the molecular level, showing, for example, that the rate of transcription of the major histocompatibility complex genes is enhanced at the transcriptional level (reviewed by Rossi, 1985). It is now clear that there are at least 25 genes in susceptible cells whose rate of transcription is increased by exposure of the cells to interferon.

Over the last few years it has become clear that cells can be induced to undergo profound changes by addition of the so-called growth factors, and we are accustomed to the wide ranging affects of this group of hormone type molecules. Should interferon be added to this list? And if so where does the interferon come from? Not from virus infection, since that would imply an obligatory role of virus infection in triggering such changes. However, it is known that a number of non-viral substances can induce interferon and there are persistent and increasingly convincing reports of the presence of small amounts of endogenous interferon in whole animals (Bocci, 1985). There are also reports of the spontaneous production of interferon by differentiating systems and a recent report shows that if such interferon is neutralised by the addition of the appropriate antibody, the differentiation process does not go to completion (Yorden *et al.*, 1984). It is, therefore, tempting to speculate that interferon may be one of a group of substances which determine the later stages of differentiation. Its inhibitory effect on those systems which are triggered by other growth factors could be seen as a reflection of counteracting systems, and its affect on terminal differentiation as a type of autocatalysis.

That is, interferon produced by cells which have travelled through the early stages of cell differentiation are exposed to inducers from an as yet unidentified source, and this interferon is essential for the later stages. Certainly the differentiation of NK cells as a response to interferon treatment has been interpreted in this way. It is clear that such later stages would not be stimulated until the cells themselves become competent to respond to interferon, and this process appears to occur about one third of the way through embryonic development. It is still not clear why the stages of early differentiation including the critically important stages of determination should not be affected by interferon. But it is clear that the genome of such early differentiating systems is controlled in such a way as to neither make nor respond to interferon.

Thus, we can take Alick Isaac's story a little further. His early observations have held up and have been explored in a much more sophisticated system, but still leave many questions to be resolved. However, the evidence accumulating over the last few years has strengthened the view that interferon may indeed play a role in the later stages of differentiation of a wide variety of cells. It is, of course, not alone in doing this and it looks as if through our investigation of the interferon system, we have stumbled into an area which will provide many more years of fascinating work. This has been perhaps one of the most enduring qualities of the interferon system; no longer is it, as it was in the early 60s, an obsession of a few scientists absorbed with what many saw to be a peripheral and rather trivial research area. The importance of the interferon system does not rest on solely whether it turns out to be a clinically useful agent against viruses or cancer; in my view its importance will remain as a lever to understand a little more of that vast network of regulated gene expression that lies behind both the formation and the activity of differentiated cells.

**References**

AGUET, M., GRESSER, I., HOVANESSIAN, A.G., BANDU, M.-T., BLANCHARD, B. & BLANGY, D. (1981). Specific high affinity binding of iodine-125 mouse interferon to interferon resistant embryonal carcinoma cells in vitro. *Virology*, **114**, 585.

BARLOW, D.P., RANDLE, B.J. & BURKE, D.C. (1984). Interferon synthesis in the early post-implantation mouse embryo. *Differentiation*, **27**, 229.

BARON, S. & ISAACS, A. (1961). Mechanisms of recovery from viral infection in the chick embryo. *Nature*, **191**, 97.

BOCCI, V. (1985). The physiological interferon response. *Immunology Today*, **6**, 7.

BURKE, D.C., GRAHAM, C.F. & LEHMAN, J.M. (1978). Appearance of interferon inducibility and sensitivity during differentiation of murine teratocarcinoma cells *in vitro*. *Cell*, **13**, 243.

CANTELL, K., VALLE, M., SCHAKIR, R., SAUKKONEN, J.J. & UROMA, E. (1965). Observations on production, assay and purification of chick embryo interferon. *Ann. Med. Exp. Fenn.*, **43**, 125.

COVENEY, J., SCOTT, G., KING, R., BURKE, D.C. & SKUP, D. (1984). Changes in the conformation of the interferon $\beta$ gene during cell differentiation and interferon induction. *Biochem, Biophys. Res. Commun.*, **121**, 290.

GROSSBERG, S.E. & MORAHAN, P.S. (1971). Repression of interferon action: induced differentiation of embryonic cells. *Science*, **171**, 77.

ISAACS, A. & BARON, S. (1960). Antiviral action of interferon in embryonic cells. *Lancet*, **ii**, 946.

KRAUSE, D., SILVERMAN, R.H., JACOBSEN, H., LEISY, S.A., DIEFFENBACH, C.W. & FRIEDMAN, R.M. (1985). Regulation of ppp $(A2'p)_nA$ dependent RNase levels during interferon treatment and cell differentiation. *Eur. J. Biochem.*, **146**, 611.

MORAHAN, P.S. & GROSSBERG, S.E. (1970*a*). Age-related cellular resistance of the chicken embryo to viral infections I. Interferon and natural resistance to myxovirus and vesicular stomatitis virus. *J. Infect. Dis.*, **121**, 615.

MORAHAN, P.S. & GROSSBERG, S.E. (1970*b*). Age-related cellular resistance to viral infections II. Inducible resistance produced by influenza virus and *Esherichia coli*. *J. Infect. Dis.*, **121**, 624.

ROSSI, G.B. (1985). Interferon and cell differentiation. *Interferon*, **6**, p. 31. Academic Press: New York.

SEHGAL, S., LEVINE, A.J. & KHOIRY, G. (1979). Evidence for nonspliced SV40 RNA in undifferentiated terato carcinoma cells. *Nature (London)*, **280**, 335.

SILVERMAN, R.H., KRAUSE, D., JACOBSEN, H., LEISY, S.A., BARLOW, D.P. & FRIEDMAN, R.M. (1983). 25-A-Dependent RNase levels vary with interferon treatment, growth rate and cell differentiation. In *The Biology of the Interferon System*, DeMaeyer, E. & Schellekens, H. (eds) p. 189. Elsevier: Amsterdam.

SLEIGH, M.J. (1985). Virus expression as a probe of regulatory events in early mouse embryogenesis. *Trends in Genetics*, **1**, 17.

WOOD, J.N. & HOVANESSIAN, A.G. (1979). Interferon enhances 2-5A synthetase in embryonal carcinoma cells. *Nature*, **282**, 74.

YORDEN, A., SHURE, GOTTLIEB, H., CHEBATH, J., REVEL, M. & KIMCHI, A. (1984). Autogenous production of interferon-$\beta$ switches on HLA genes during differentiation of histiocytic lymphoma U937 cells. *EMBO J.*, **3**, 969.